# Effect of Prosopis Juliflora Thorns on Mechanical Properties of Plastic Waste Reinforced Epoxy Composites

**DOI:** 10.3390/polym14071278

**Published:** 2022-03-22

**Authors:** Sakthi Balan Ganapathy, Aravind Raj Sakthivel, Mohamed Thariq Hameed Sultan, Farah Syazwani Shahar, Ain Umaira Md Shah, Tabrej Khan, Tamer A. Sebaey

**Affiliations:** 1 Department of Manufacturing Engineering, School of Mechanical Engineering (SMEC), Vellore Institute of Technology, Vellore 632014, Tamil Nadu, India; sakthibala33@gmail.com; 2 Department of Aerospace, Faculty of Engineering, Universiti Putra Malaysia, Serdang 43400, Malaysia; farahsyaz93@yahoo.com (F.S.S.); ainumaira91@gmail.com (A.U.M.S.); 3 Laboratory of Biocomposite Technology, Institute of Tropical Forestry and Forest Products (INTROP), Universiti Putra Malaysia, Serdang 43400, Malaysia; 4 Aerospace Malaysia Innovation Centre (944751-A), Prime Minister’s Department, MIGHT Partnership Hub, Jalan Impact, Cyberjaya 63000, Malaysia; 5 Engineering Management Department, College of Engineering, Prince Sultan University, Riyadh 12435, Saudi Arabia; tkhan@psu.edu.sa; 6 Mechanical Design and Production Department, Faculty of Engineering, Zagazig University, Zagazig 44519, Sharkia, Egypt

**Keywords:** waste plastics, fillers, Prosopis juliflora, thorn powder, abrasive wear resistance

## Abstract

Plastics are unavoidable at this times, putting our planet in danger. The Prosopis juliflora (PJ) thorns are collected, processed, and powdered. The mechanical characteristics of these powders are examined when combined with polymer composites. Pores are the main cause of moisture input, hence using powder filler materials reduces the number of pores in the composite, increasing water resistance. The composites are made by altering three parameters: waste plastic content, filler powder composition, and chemical treatment. It was discovered that the integration of thorn powder increased the wear resistance. The composites were tested in accordance with ASTM standards, and the results were optimized. Based on the results, composite specimens were created and tested for validation.

## 1. Introduction

Due to the plastic thrash, the land, water, and air are polluted [1]. If the plastics are dumped in the landscapes, they will block the percolation of rainwater into the land which leads to a decrease in the groundwater levels. If the plastics are thrown into water bodies, it leads to leaching, and the water gets polluted. We cannot burn the plastics, as it would release harmful gases into the atmosphere. To avoid the degradation of the earth, the used plastics have to be reused in some way. In the growing world, the need for alternate materials is increasing. At the same time, lots of landscapes are occupied by Prosopis juliflora plants, which makes it difficult to cultivate crops and also it absorbs the water and nutrients from the land and makes it uncultivable [2,3]. A lot of research works are undergoing to use these plants effectively for various applications. Kailappan et al. produced the activated carbon from Prosopis juliflora using a chemical method and proved it can be used in oil, food, and pharma industries [4]. As the plastics are used in this composite, it can be used in automobile, marine applications where lightweight and less corrosive materials are required. Plastics have moisture repelling property and the Prosopis juliflora thorn powder provides hardness to the composites and also it fills the minute pores produced during the production of composites.

When Saravanakumar and his colleagues analysed the parameters of the fibres, they found that they had an average microfibril angle of 10.64 and utmost elongation strength of 558 MPa. It was found that the aspect ratio of the PJ fibres had a significant impact on the mechanical qualities of the finished product. A fibre aspect ratio of 136 and fibre loading of 23.53 wt. percent were found to yield the best mechanical properties. [5]. The tensile and thermal properties of treated PJ fibres were shown to be superior to those of untreated fibres in a study by Madhu et al. [6].

Structural applications may benefit from the properties of PJ fiber- and glass-fiber reinforced polymer composites, according to Manoj Kumar et al. [7]. Due to the presence of lignin, researchers led by Luis Valencia found functional nanomaterials with a mean radius of 10 nm and a length of 150 nm in the PJ [8]. Filler-reinforced epoxy composites can improve mechanical properties by as much as 45 percent, according to research by Santhosh et al., who studied the morphology and properties of PJ and RH—reinforced composites [9]. PJ ash powder can be used as a substitute for cement up to 20%, according to Parthiban Kathirvel et al., and they were able to attain the same strength for their newly designed concrete [10]. An onion-like porous carbon made from the PJ has been advocated as an efficient electrode material by Sathyanarayanan Shanmugapriya and his colleagues [11].

The novel composites’ mechanical, tribological, and water absorption properties must be thoroughly investigated. It was discovered by Sakthi Balan et al. that adding 30 wt percent of waste plastic particle to jute fibre and waste plastic-filled composites resulted in high resistance to water absorption [12]. Water absorption and tribological properties were increased by the inclusion of filler and fibres to the composite, which was made of plastic waste, fiber glass, and silica sand fillers. It has been reported that the thermal and mechanical characteristics of epoxy composites manufactured with 20% fibres and NaOH treatment have been improved [13] by Arthanarieswaran and colleagues. Using dates palm seeds and glass fibre reinforced polymer composite, Heba I. Elkhouly et al. demonstrated an increase in the composite’s wear resistance and toughness. Tapas by Priyadarshi Tapas Ranjan Swain et al. made a composite out of jute fibres and studied how it wore. The wear resistance has been modified by the chemical treatment [14]. They observed that the abrading distance was the most important element in determining the wear of waste silk fiber-reinforced epoxy composites [15], followed by the loading of fibers.

Taguchi is an effective strategy for designing experiments. Materials scientists use it to their advantage when examining the effects of various process variables. Polymer-based composites are being made using the Taguchi method. Boron nitride reinforcement of Nylon composite was studied by Shiva Kumar and Chennakesava Reddy using the Taguchi approach and it was found that the composite’s wear resistance was improved by the addition of boron nitride [16]. It was proposed by Wahid Ferdous et al. that the bond length and thickness for higher strength be studied using Taguchi design in polymer-based composites [17]. Polymer composites with various reinforcements such kevlar, carbon, and glass fibers were subjected to the Taguchi technique by Karthik et al. in order to optimize the wear parameters [18]. They found that the hybrid composites had improved wear behaviors. As a result of the Taguchi approach and ANOVA, Siva Prasad and Chaitanya were able to optimize the drilling parameters for the GFRP composites [19].

Natural fibers have gained popularity and are beginning to supplant synthetic fibers, owing to their contribution to sustainable practices. As a result of environmental, social, and economic development, numerous industries have altered their manufacturing processes, materials, and procedures in order to ensure a sustainable future. While natural fibers have significant disadvantages, they can be overcome with appropriate chemical treatments and fiber processing. Numerous goods composed of natural fibers are developed and used in sports, electronics, and musical instrument manufacturing [20]. Natural fibers exhibit comparable wear resistance to synthetic fibers. The wear resistance of natural fibers can be increased by reinforcing them with synthetic fibers [21]. Recent research has concentrated on green fillers such as date seed powders, coconut and cashew nut shell powders, and rice and maize husks. Natural fibers and fillers are used in composites because they are inexpensive, widely available, biodegradable, recyclable, and lightweight [22]. Researchers are becoming more interested in starch-based bioplastics due to their environmentally beneficial characteristics. Starches have been isolated from a variety of plants, including PJ plants, and bio composites have been constructed and tested for mechanical properties and biodegradability. The results indicated that composites might be used as a substitute material in the packaging industry [1].

Plastic trash and PJ thorn granules are both included into the polymer matrix in this study. The motive of this work is to investigate the influence of chemical treatment, the amount of waste plastics, and PJ thorn powders on the composite’s hardness and moisture absorption and wear capabilities using the Taguchi technique.

## 2. Experimental Details

### 2.1. Materials

The scraped compact discs are employed as reinforcement in this composite fabrication, and most of them are constructed of polycarbonate. The decomposition of polycarbonate can take hundreds of years, yet it is possible. The reinforcements are placed in a matrix made of epoxy resin. Araldite AW106 grade resin and HV 953U hardener are utilized in this. Epoxies have features that distinguish them from other resins, and adjustments can be made to meet our specifications. Epoxies have superior mechanical qualities, including increased thermal stability, wear and chemical resistance, and resistance to ageing caused by environmental factors [22]. In order to improve a composite’s specific characteristics and traits, fillers are incorporated into the material. Fillers in this work include PJ thorn powders. In general, PJ plants cause a wide range of issues for both humans and the environment. As a result, the PJ plant’s thorns are harvested, dried for a period of time, and ground into a powder. The waste compact discs were collected from Vellore, Tamilnadu, India, the resin and hardener were purchased from Ayishwarya polymers, Coimbatore, Tamilnadu, India, and the PJ thorn powders were made manually by collecting the PJ thorns from nearby areas in and around Vellore, Tamilnadu, India, drying them in the sun, and then powdering them using a mixer grinder. Finally, before use, the PJ powder is sieved and processed. Once these powders have been chemically treated, they can be used as reinforcements and the matrix, as they have been neutralized of their characteristics.

### 2.2. Fabrication Method

Initially, the raw materials are collected like waste used plastics. The collected plastics are then separated according to their grades, cleaned, dried, and then they are crushed into particulates. Then the filler powder is made by collecting the PJ thorns, drying and then they are chemically treated with alkalis and silanes for one hour. The NaOH solution (5% *w*/*v*) is used for alkali treatment and Triethoxy vinyl silane (5% *v*/*v*) is used for silane treatment [23]. In both cases, the thorn powders are dipped into the chemicals for an immersion time of one hour. After the alkali treatment, the thorns are washed with HCL solution to make their pH value-neutral and the silane treated thorns are washed with de-ionized water and then dried. Then they are crushed and made into powders. The resin and the hardener are mixed in the proper ratios and the composite is made by the spray layup method. Spray layup has some added advantages to the hand layup method and the defects are low when compared to other manufacturing techniques. As the plastic particulates and PJ thorn powder can be dispersed through air medium, spray layup is preferred. Initially, the resin was applied and then the reinforcement and the fillers are loaded in the spray gun, and with the help of compressed air as a medium, the particles are sprayed evenly on the resin surface. This ensures the even spread of the fillers.

### 2.3. Mechanical Properties Examination Methods

The Vickers micro hardness tester manufactured by Fuel Instruments & Engineers Pvt. Ltd., Ichalkaranji, Maharashtra, India, is used to measure micro hardness, and the ASTM E10 standards are followed for the method. Test specimens are imprinted in Vickers hardness testers with 2.5-mm diameter balls of 10 kg of force. The specimen was indented in numerous places, and the average value is used to get the end result. To compute the average values, the diagonals of the cavity generated by pressing a diamond pyramid, are used.

The elongation of specimens on a UTM was measured using the ASTM D 3039-76 standard testing procedure. In this experiment, we used a UK-made H10KS model from Tinius Olsen, Redhill, UK. Crosshead speed and strain rate are maintained at 5 mm/min. Water jet cutting minimizes the formation of micro cracks compared to other cutting procedures, the specimens are cut using this method.

ASTM D570 standards were used to conduct the water intake examination for composites. As a precautionary measure, sticky tape is used to cover the specimen’s sides. A scale is used to weigh the samples in advance of testing, and the results are recorded. Test pieces are then submerged in water for 24 h for examination. Afterward, the test pieces are taken out, patted dry, and weighed to determine the weight gain that occurred during the testing period. The percentage of water consumed will be determined by comparing the pre- and post-testing results. The moisture percentage is calculated using the following equation [12].

MA% = (W_2_ − W_1_)/W_1_ × 100, (1)

where,

MA% = Moisture absorption percentage
W_1_ = Sample weight before the experiment in gms
W_2_ = Sample weight after the experiment in gms

The abrasive wear of the composite is measured using a pin on a disc wear testing equipment. The specimen was cut in accordance with the specifications of ASTM G99-05. Wear testing equipment supplied by DUCOM instruments, Peenya Industrial Area, Bengaluru, Karnataka, India, has a maximum wear track diameter of 135 mm and a maximum disc speed of 2000 rpm, which was used for the testing. A frictional force of 200 N and a load of 20 Kg are the maximums that can be applied. An abrading distance of 420 m and a load of 10 Newtons are applied to the specimen, which measures 5930 mm in size. With the use of a cantilever unit, the specimen is put into the holder and held there by the disc. Before loading the specimen, it is weighed, and then the test is performed. The samples will be weighed before and after the tests. The density is used to compute the wear volume. In order to calculate the wear rates, Equation (2) is used [24].

Wear rate (K_s_) = Differences in wear volumes in mm^3^/(Normal load in N) × (Abrading distance in m) (2)

### 2.4. Optimization Technique

Optimization was done on three concepts based on our requirements. Larger is better, nominal is better, and smaller is better are the three concepts under which the optimization was done [5]. In our case for water absorption, the percentages must be low, so smaller is better concept is used [25]. For micro hardness, larger is better concept is used as normally polymer composites have good tensile and bending characteristics [6]. The hardness is required in some cases where it is subjected to wear and abrasion applications. The influencing parameters such as the composition of waste plastics, PJ thorn powder, and type of chemical treatment are chosen and the most influencing parameter which affects the hardness and the water intake properties are found out. The optimum values of these parameters are also found and the validations were done to find out the nearness of the results with the predicted results. The design for specimen production is obtained through Taguchi’s L27 full factorial experimental design, then the prepared specimens are tested and the test results are fed to the software and run for getting the optimum values [21]. In full factorial design the number of experiments is more, so that the results of the full factorial will be more accurate than the fractional factorial. The process parameter chosen and its stages were indicated in Table 1. The experimental results are given in Table 2.

## 3. Results and Discussion

### 3.1. Hardness Analysis

**Figure 1:**
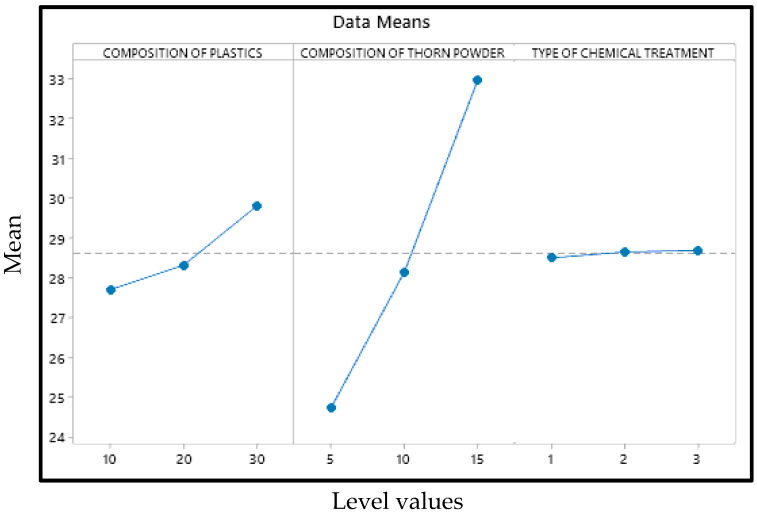
Effects chart for Hardness.

**Figure 2:**
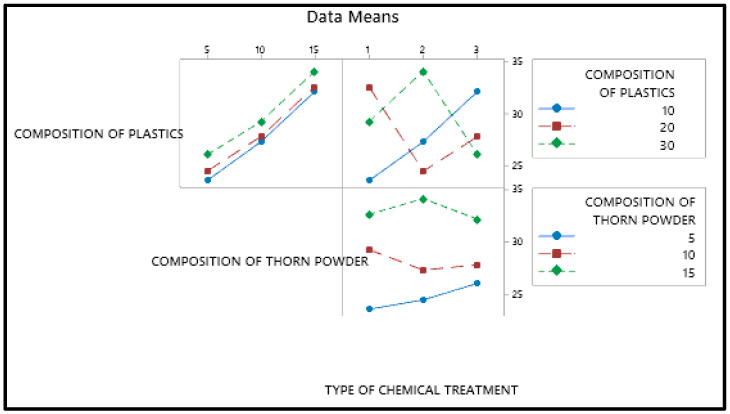
Interaction plot for SN ratios for Hardness.

**Figure 3:**
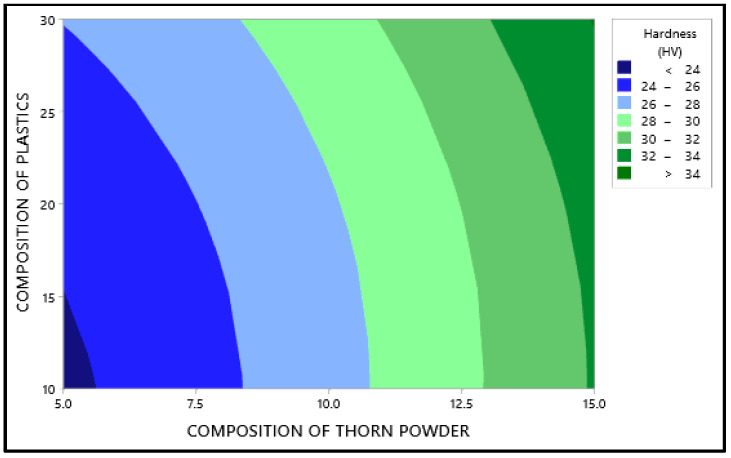
Contour plot for Maximum Hardness.

The primary effect map for the composite samples’ hardness may be seen in Figure 1 with mean of the output factor in the Y axis and values of the levels in the X axis. Figure 1 shows that the amount of plastic and the type of chemical treatment have the less impact on hardness, than composition of PJ thorn powder. In this case, the chemical therapy has the least impact. The harder the final product is obtained as a result of the increased filler content. The fillers in the matrix provide resistance, which enhances hardness [26]. Another component that improves the composite’s properties is the bonding between the matrix and filler [27]. Not all materials will adhere to the resin. Fillers are distributed uniformly throughout the composites; however there is some variance in hardness due to the substance with which the indenter makes contact. At times, the indenter comes into touch with the filler, and at other times, it comes into contact with the resin or plastic particles, resulting in a range of hardness values. To ensure accuracy, readings are taken at multiple locations and the average values are used as the final result. Due to the hydrophobic nature of natural fillers and fibers, they will have low wettability. This renders them incompatible with resins, and following a chemical reaction, the hydrophobic nature of the fillers is converted to hydrophilic, increasing their wettability. Figure 2 and Figure 3 illustrate the interaction and contour plot for the hardness of the material. Using a contour map, hardness enhancement by the plastics and thorn powder composition inclusion is clearly seen. It is possible to see the ANOVA findings in Table 3. Thorn powder composition is determined to have the greatest impact on hardness properties, according to ANOVA results.

**Figure 4:**
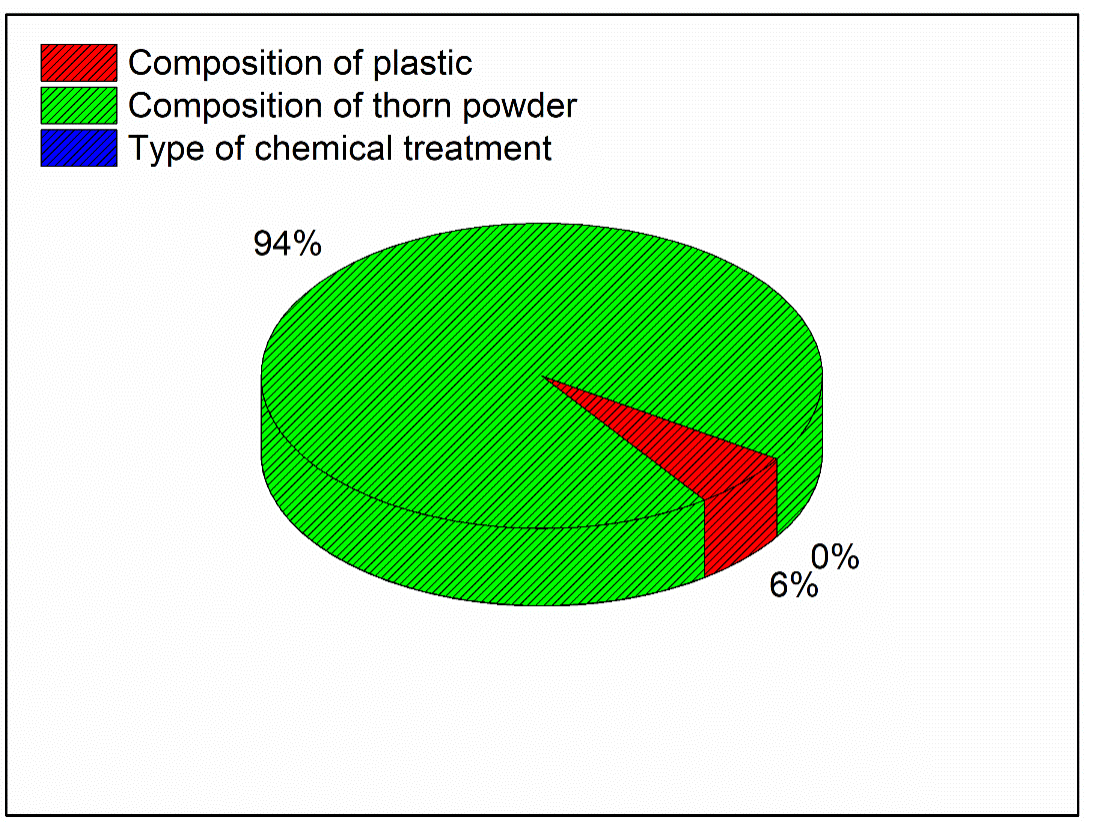
Contribution plot for Maximum Hardness.

In addition, the composition of plastics as well as the chemical treatment has least effect on the hardness of the material. According to the model summary table, the R-square number indicates how near the findings are to the mean values; an R-square value of 98.53 percent indicates that the results are extremely close to the mean values. The contribution plot for the hardness is depicted in Figure 4. The contribution plot is created by plotting the F-values from the ANOVA results together. The F-value indicates that the addition of thorn powder has the most influence on hardness among all of the components, and the contribution plot suggests that the addition of plastics has the second greatest influence on hardness. As seen in Equation (3), the regression equation for hardness is a linear relationship.

Regression equation for Hardness (HV) = 18.057 + 0.1058 Composition of plastics + 0.8250 Composition of thorn powder + 0.094 type of chemical treatment(3)

### 3.2. Water Absorption Property

**Figure 5:**
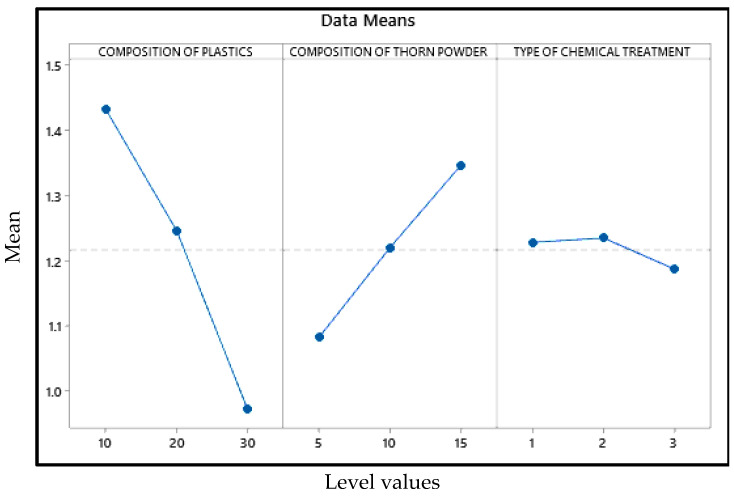
Effects plot for water absorption.

**Figure 6:**
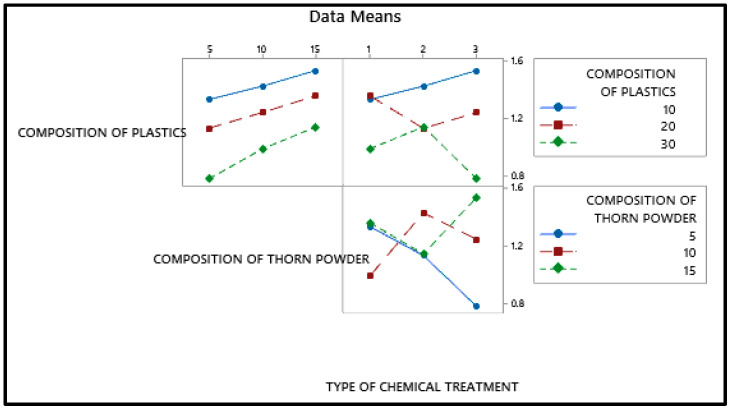
Interaction plot for water absorption.

**Figure 7:**
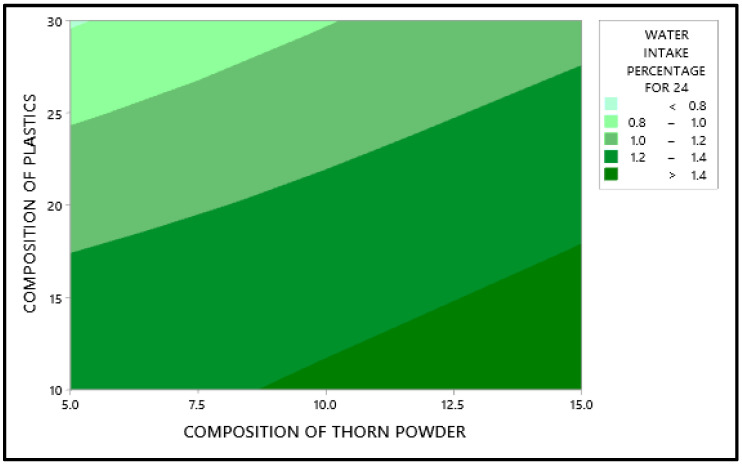
Water intake test result through contour graph.

**Figure 8:**
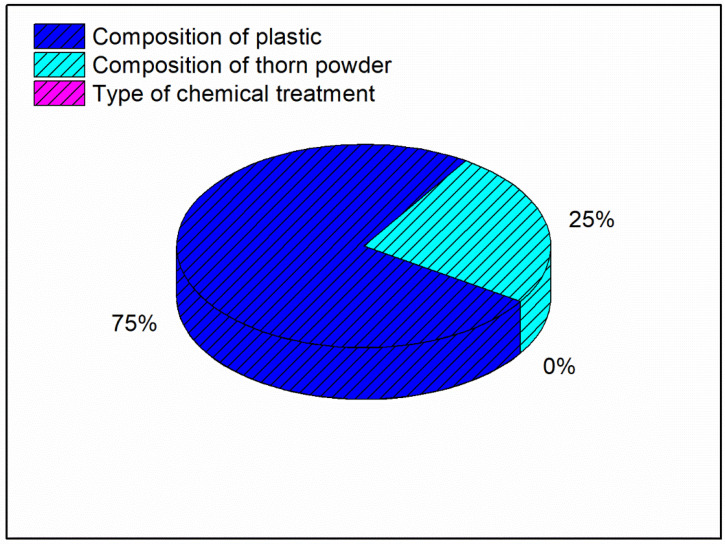
Contribution plot for water absorption.

Figure 5 shows that the plastic composition should be 30%, the inclusion of thorn powder should be 5%, and it should be silane-treated. In Figure 5, the mean values of the output factor (water absorption percentage) is in the Y axis and the level values are in the X axis. The water absorption property of waste plastic is more strongly influenced by its composition. As a general rule, water cannot be absorbed by plastic. As a result, adding plastic to the composite makes it more water resistant [28]. Additionally, the chemical bonds that bind the monomers are strong enough that plastics take years to breakdown. Plastics are constructed in such a way that water cannot percolate into them. Additionally, the filler powders aid in resisting water percolation, albeit only to a certain amount [29]. If they come into direct touch with water, they dissolve and, in certain situations, cause the composites to bulge. Figure 6 depicts the interdependence of the variables, showing how they interact with one another. It demonstrates that the addition of waste plastics and the addition of thorn powder are not interdependent. Figure 7 shows the contour plot, which shows that for the least amount of water absorption, 30 weight percent of plastics and 5 weight percent of thorn powder are added. Table 4 shows that the addition of waste plastic particles has the greatest impact on the composites’ ability to absorb moisture, followed by the composition of thorn powders. The water intake qualities of PJ thorn powders are unaffected by the chemical treatment that was performed on them. Nearness to the mean is indicated by the R-Square value of 97.06. The F-value from the ANOVA table was used to generate the contribution plot in Figure 8.

The regression equation for minimum water intake in 24 h = 1.45−0.023056 Composition of plastics + 0.02644 Composition of thorn powder − 0.02056 types of chemical treatment(4)

### 3.3. Tensile Strength

**Figure 9:**
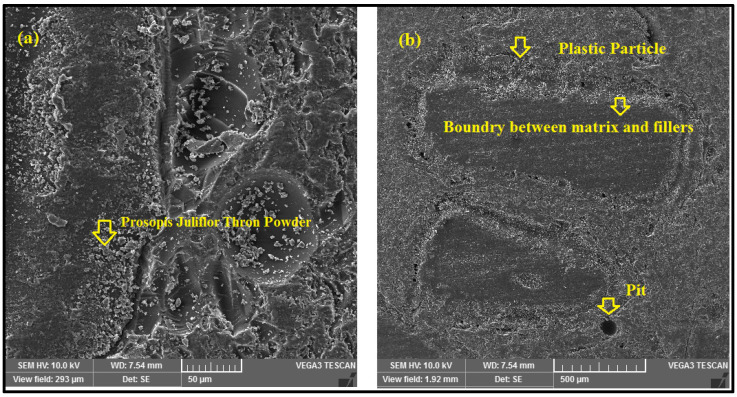
(**a**). SEM image with 50 µm magnification (**b**). SEM image with 500 µm magnification.

**Figure 10:**
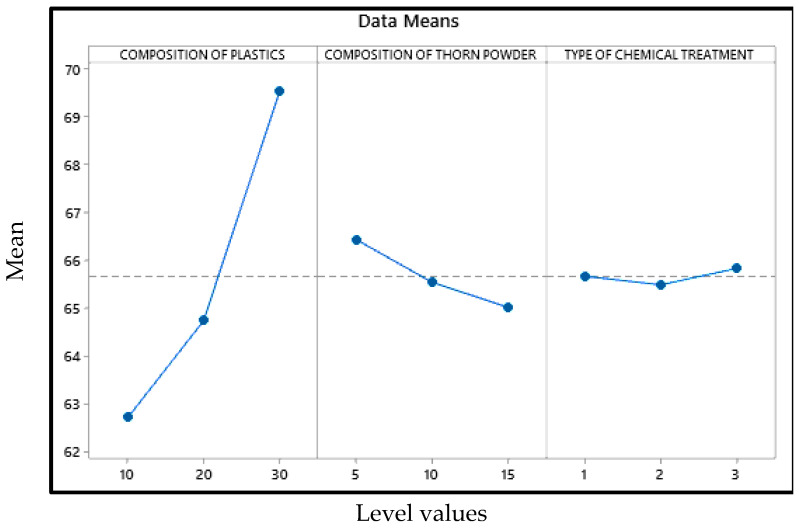
Effects graph for tensile strength.

According to the results of the optimization, in order to achieve optimum tensile strength, the amount of plastics added must be maximized, and the amount of thorn powder added must be 5 weight percent, with the treatment of the thorn powder having little effect. The addition of greater volumes of waste plastic improved the elongation strength. It is possible to achieve an improvement in tensile strength by improving the bonding between the matrix and the reinforcement [30]. The tensile results demonstrate that the plastics introduced as reinforcement have a stronger bonding to the matrix. There will be gaps between the reinforcements and the matrix in some locations due to the resin shrinking during curing, which will result in the composite failing when the load is applied. However, in this scenario, the problem is resolved, since the fillers cover the gaps and improve the composite’s performance under pressure. As shown in Figure 9, the scanning electron microscope pictures were captured after the sample had ruptured, with the emphasis being on the ruptured area of the sample. SEM images will show the wear and erosion mechanisms that happened during wear testing [21]. The key effects plot for tensile strength is depicted in Figure 10 in which the tensile strength mean is mentioned in the Y axis and their levels are indicated in X axis.

**Figure 11:**
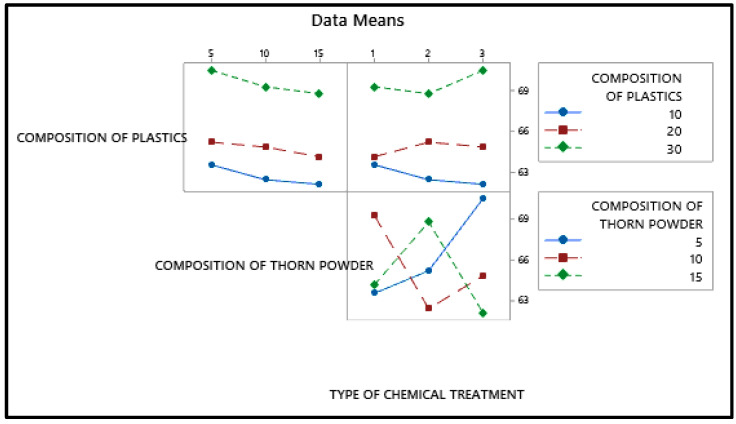
Interaction plot for Maximum tensile strength.

**Figure 12:**
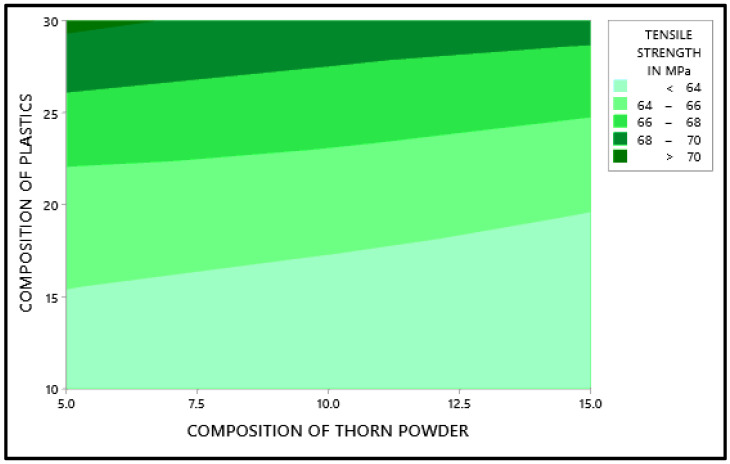
Tensile strength results through contour graph.

**Figure 13:**
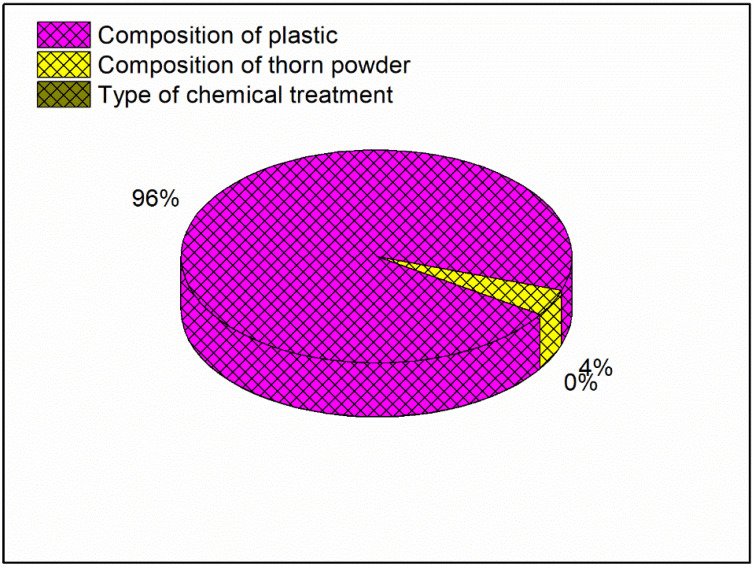
Contribution plot for Maximum tensile strength.

Particulate plastics are visible; thorn powder is equally dispersed on top of the composite. The fiber-matrix interface reveals improved adhesion between plastics and fibers. Due to the detachment of plastic particles, pits can form on the surface. Figure 11 shows the relationship between the input and output parameters through an interaction plot. A3B1C3 are the input elements needed to achieve maximal tensile strength. In Figure 12, contour plot likewise mirrors the main effect plot’s results. To see the tensile strength of the plastic and thorn powder mixture, look at the graph in Figure 13. Plastic particles make up most, while thorn powder has little effect on tensile characteristics which is seen in Table 5. There is almost a 95 percent confidence level in the model is seen based on the R-Squared value. The results are in agreement with the findings of earlier researchers [6,7]. Figure 13 displays the tensile strength contribution plot.

Regression equation for Maximum tensile strength = 60.079 + 0.3419 Composition of plastics − 0.1421 Composition of thorn powder + 0.082 type of chemical treatment(5)

### 3.4. Abrasion Wear Behavior

**Figure 14:**
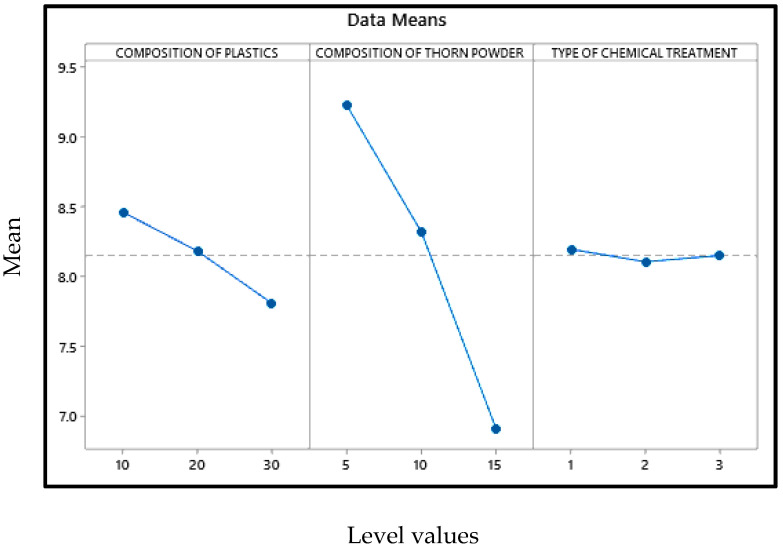
Main effect plot for minimum wear rate.

**Figure 15:**
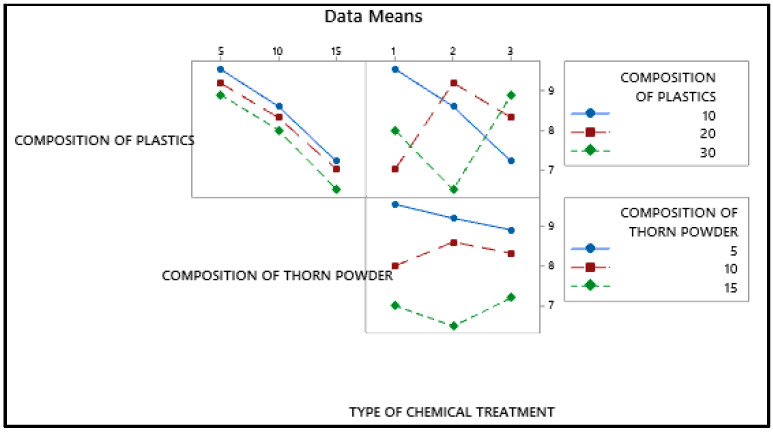
Interaction plot for minimum wear rate.

**Figure 16:**
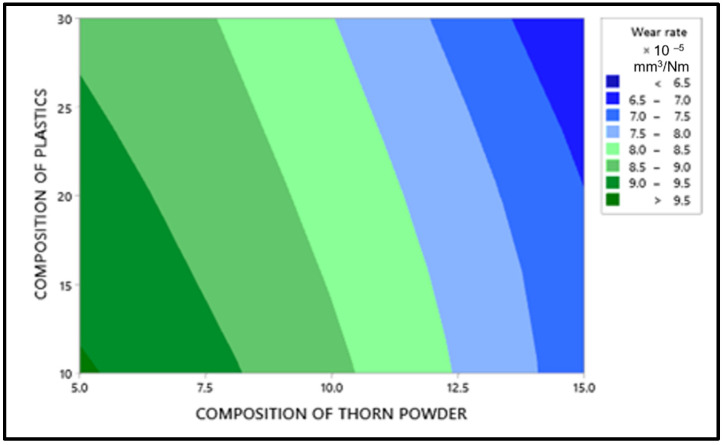
Wear rate results through contour graph.

**Figure 17:**
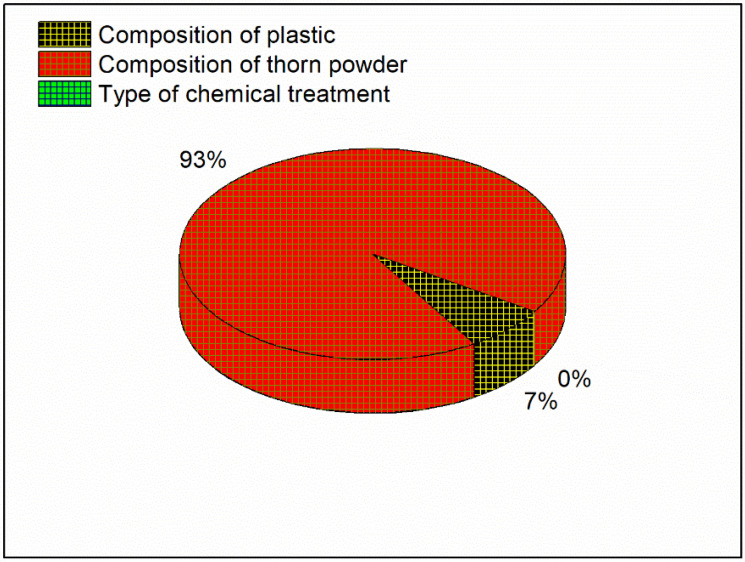
Contribution plot for Minimum wear rate.

For the lowest wear rate, the main effects plot is shown in Figure 14 in which the mean value of the wear rate is mentioned in Y axis with their levels in X axis. Porosity reduction due to the inclusion of thorn powder is the important factor in deciding composite’s wear resistance. However, the amount of waste plastic and the type of chemical treatment do not have a significant impact. The increase in hardness is due to the increased amount of thorn powder [14]. Hardness has a direct correlation to wear resistance [31]. Wear resistance is improved by increasing the amount of hardness. The wear-resistant is boosted by the thorn powder. Results for several polymer-based composite fillers have been published by others [14,15,32,33]. The interaction plot for the lowest wear rate can be seen in Figure 15. Plastic and thorn powder have the strongest interactions. For a clearer picture of the wide range of possible reactions, see the contour plot in Figure 16. There must be 30 percent plastics and 15 percent thorn powder added in order to achieve a minimum wear rate of 0.05%. The main effect plot for minimal wear rate is shown in ANOVA Table 6, thorn powder contributes more to wear resistance than other additives. Thorn powder contributes 93 percent of the total contribution, while plastics supply 7 percent. The wear resistance of the composite is not affected by the chemical treatment. The composites’ wear rate contribution plot is shown in Figure 17. Equation (4) contains the regression equation, and an R-Squared value of 98.39% shows that the experimental responses are closer to the mean values.

Regression equation for Minimum rate of wear = 11.168 − 0.03261 composition of plastics − 0.232 composition of thorn powder − 0.0222 type of chemical treatment(6)

## 4. Conclusions

PJ fibers are mixed with a variety of natural and synthetic fibers to create enhanced-property hybrid composites. Natural fibers’ high strength-to-weight ratio, longevity, and inexpensive cost make them an excellent choice for polymer composites. Natural fiber composites are widely used in defense, automotive, and marine applications. A composite is made and tested using glass fiber and PJ in powder form. The results indicate that adding 6% PJ powder to glass fiber composites leads in increased impact and compressive strengths, as well as increased hardness [34].

In this study, the composite was created by combining waste plastic particles with Prosopis juliflora thorn powder according to Taguchi’s full factorial design and laying it out by spraying. The trials are carried out in accordance with ASTM standards, and the results are entered into a software programme for further optimization. According to the optimal values, the additions of thorn powder improves the hardness and wear resistance property, and the inclusion of waste plastics improves the resistance to moisture absorption and the tensile properties of the material. A material with a high hardness will have a higher resistance to wear due to friction and abrasion. The use of fillers increases the composite’s hardness, which is reflected in the composite’s wear rate [35].

It is necessary to have a 30 weight percent composition of plastics, a 15 weight percent addition of thorn powder, and it must be silane treated in order to get maximum hardness. In order to get the lowest possible water intake while maintaining the highest possible tensile property, 30 weight percent waste plastic particles and 5 weight percent thorn powders must be incorporated. Due to the natural nature of the filler powder, its inclusion must be kept to a minimum to ensure optimal resistance to moisture absorption. Even if chemically treated, they will lose their hydrophobic properties in extreme circumstances and for extended periods of time, allowing water to permeate through the composites. Overall, chemical treatment had little effect on the hardness, tensile strength, or moisture intake characteristics of the material. Finally, in order to get the lowest possible abrasion wear, the maximum amount of polymers and thorn powder should be used. The validation tests are carried out for hardness in accordance with the projected levels, and it is discovered that the error value falls below the acceptability criteria.

## Figures and Tables

**Table 1:** Process variables and their various stages.

S. No.	Variables	Units	Stages		
			1	2	3
1	Particulates of waste plastics	Wt%	10	20	30
2	Prosopis juliflora thorn powder	Wt%	5	10	15
3	Chemical treatment	-	Nil	NaOH (Alkali)	Triethoxy vinyl silane

**Table 2:** Full factorial inputs and outcomes.

Trail No	Process Parameters			Hardness (Hv)	Tensile Strength (MPa)	Water Intake Percentage for 24 h	Wear Rate × 10^−5^ mm^3^/Nm
	Plastic Particulates (Wt%) (A)	Thorn Powder (Wt%) (B)	Type of Chemical Treatment (C)				
1	10	5	1	23.6	63.56	1.35	9.55
2	10	5	1	23.5	63.55	1.32	9.55
3	10	5	1	23.7	63.56	1.33	9.58
4	10	10	2	27.2	62.44	1.44	8.6
5	10	10	2	27.5	62.44	1.41	8.62
6	10	10	2	27.3	62.46	1.43	8.61
7	10	15	3	32.1	62.12	1.53	7.2
8	10	15	3	32.4	62.11	1.54	7.22
9	10	15	3	32	62.09	1.54	7.2
10	20	5	2	24.8	65.22	1.15	9.2
11	20	5	2	24.6	65.22	1.12	9.21
12	20	5	2	24.1	65.21	1.13	9.21
13	20	10	3	27.8	64.87	1.23	8.33
14	20	10	3	27.9	64.85	1.24	8.32
15	20	10	3	27.8	64.86	1.26	8.33
16	20	15	1	32.5	64.15	1.36	7.01
17	20	15	1	32.6	64.12	1.35	7.02
18	20	15	1	32.8	64.13	1.37	7
19	30	5	3	25.9	70.54	0.8	8.91
20	30	5	3	26.1	70.51	0.79	8.91
21	30	5	3	26.2	70.52	0.75	8.92
22	30	10	1	29.1	69.3	0.97	8
23	30	10	1	29.2	69.31	0.96	8.01
24	30	10	1	29.5	69.31	1.04	8.02
25	30	15	2	34.1	68.79	1.18	6.5
26	30	15	2	34.2	68.8	1.15	6.49
27	30	15	2	34.05	68.79	1.1	6.5

**Table 3:** Hardness ANOVA.

Source	DOF	Adjacent SS	Adjacent MS	F-Values	*p*-Values
Regression	3	326.603	108.868	514.7	0
Composition Of Plastics	1	20.161	20.161	95.32	0
Composition Of Thorn Powder	1	306.281	306.281	1448.02	0
Type Of Chemical Treatment	1	0.161	0.161	0.76	0.393
Error	23	4.865	0.212		
Lack-Of-Fit	5	4.253	0.851	25.03	0
Pure Error	18	0.612	0.034		
Total	26	331.468			

S: 0.459911, R^2^ (Adjacent): 98.53%, R^2^ (Predicted): 98.03%.

**Table 4:** Water intake results from DOE.

Variables	D.F	Adj SS	Adj MS	F-Value	*p*-Value
Regression	3	1.2791	0.426367	253.2	0
Composition of Plastics	1	0.95681	0.956806	568.21	0
Composition of Thorn Powder	1	0.31469	0.314689	186.88	0
Chemical Treatment	1	0.00761	0.007606	4.52	0.045
Error	23	0.03873	0.001684		
Lack-of-Fit	5	0.02813	0.005626	9.55	0
Error	18	0.0106	0.000589		
Total	26	1.31783			

S: 0.041035, R-Sq: 97.06%, R-Squared (Adj): 96.68%, R-Squared (Predicated): 95.98%.

**Table 5:** Tensile strength results from DOE.

Variables	D.F	Adj SS	Adj MS	F-Value	*p*-Value
Regression	3	219.608	73.203	136.59	0
Composition Of Plastics	1	210.398	210.398	392.57	0
Composition Of Thorn Powder	1	9.088	9.088	16.96	0
Type Of Chemical Treatment	1	0.122	0.122	0.23	0.638
Error	23	12.327	0.536		
Lack-of-Fit	5	12.325	2.465	20797.84	0
Pure Error	18	0.002	0		
Total	26	231.935			

S: 0.732084, R^2^: 94.69%, R^−2^ (Adj): 93.99%, R^−2^ (Pred): 92.84%.

**Table 6:** Wear rate results from DOE.

Variables	D.F	Adjacent SS	Adjacent MS	F-Value	*p*-Value
Regression	3	26.1904	8.7301	467.52	0
Composition Of Plastics	1	1.9143	1.9143	102.51	0
Composition Of Thorn Powder	1	24.2672	24.2672	1299.58	0
Type Of Chemical Treatment	1	0.0089	0.0089	0.48	0.497
Error	23	0.4295	0.0187		
Lack-of-Fit	5	0.4278	0.0856	888.4	0
Pure Error	18	0.0017	0.0001		
Total	26	26.6199			

S: 0.13665, R^−2^: 98.39%, R^−2^ (Adj): 98.18%, R^−2^ (Pred): 97.86%.

## Data Availability

Not applicable.

## Abbreviations

PJ	Prosopis juliflora
RH	Rice husk
NaOH	Sodium hydroxide
ANOVA	Analysis of Variance
GFRP	Glass fiber reinforced polymer
W/V	Weight by volume
V/V	Volume by volume
HCL	Hydrochloric acid
pH	Potential of Hydrogen
UTM	Universal testing machine
ASTM	American society for testing and materials
UK	United Kingdom
DOE	Design of Experiments
DOF	Degrees of freedom
SS	Sum of squares
MS	Mean sum of squares
F-value	Fisher’s value
*p*-value	Probability value
R^2^	Regression square
SEM	Scanning electron microscopy
